# Unraveling and Resolving the Inconsistencies in Tafel Analysis for Hydrogen Evolution Reactions

**DOI:** 10.1021/acscentsci.3c01439

**Published:** 2024-02-20

**Authors:** Chengzhang Wan, Yansong Ling, Sibo Wang, Heting Pu, Yu Huang, Xiangfeng Duan

**Affiliations:** † Department of Chemistry and Biochemistry, University of California, Los Angeles, California 90095, United States; ‡ Department of Materials Science and Engineering, University of California, Los Angeles, California 90095, , United States; § California NanoSystems Institute, Los Angeles, California 90095, United States

## Abstract

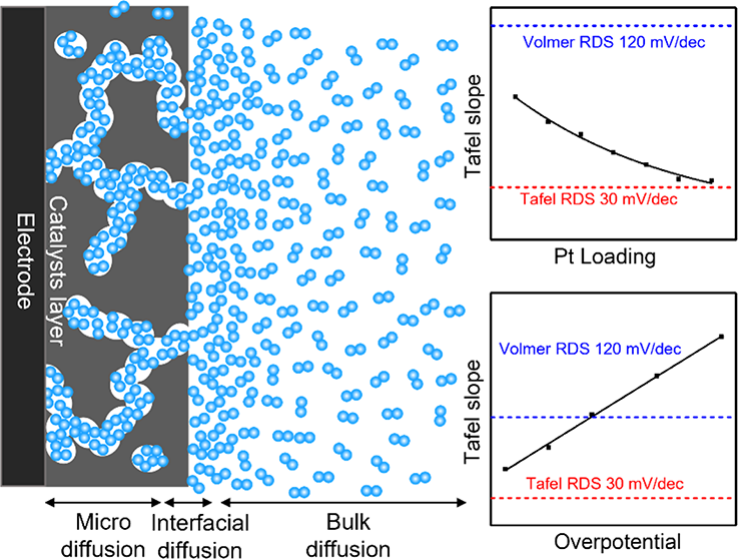

The Tafel slope represents a critical kinetic parameter for mechanistic studies of electrochemical reactions, including the hydrogen evolution reaction (HER). Linear fitting of the polarization curve in a N_2_-saturated electrolyte is commonly used to determine Tafel slopes, which is, however, frequently plagued with inconsistencies. Our systematic studies reveal that the Tafel slopes derived from this approach are loading- and potential-dependent, and could substantially exceed the theoretical limits. Our analyses indicate that this discrepancy is largely attributed to the locally trapped HER-generated H_2_ in the catalyst layer. A non-negligible hydrogen oxidation reaction (HOR) current more prominently offsets the HER current at the smaller HER overpotential regime, resulting in an artificially smaller Tafel slope. On the other hand, at the higher overpotential where the HOR current becomes negligible, the locally trapped H_2_ substantially suppresses further HER current growth, leading to an artificially larger Tafel slope. The Butler–Volmer method accounts for both the HER and HOR currents in the fitting, which offers a more reliable method for pure Pt catalysts but is less applicable to transition-metal decorated Pt surfaces with distinct HER/HOR kinetics. Our studies underscore the challenges in Tafel slope analysis and the need for strict controls for reliable comparisons among different catalyst systems.

## Abstract 2

The Tafel slopes for the hydrogen evolution reaction show distinct loading and potential dependence, which can be attributed to diffusion limitations and locally trapped H_2_ in the catalyst layer.

## Introduction

The platinum (Pt)-catalyzed hydrogen evolution and oxidation reactions (HER/HOR) represent the most extensively investigated electrochemical redox reactions for their critical relevance to the hydrogen economy. Despite their apparent simplicity, detailed mechanisms and the intriguing pH-dependent HER/HOR kinetics on the Pt surface are far from clear after decades of research. In general, there are three elementary steps that could fundamentally affect the HER/HOR process:^1,2^

(i) Volmer step:
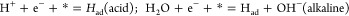
1

(ii) Tafel step:
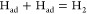
2

and (iii) the Heyrovsky step:
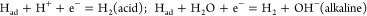
3

Tafel analysis is often used to extract HER/HOR kinetic information and guide the development of mechanistic insights.^3,4^ The Tafel slope, defined as the voltage (potential) swing required to change the electrochemical current by 1 order of magnitude, provides important information on the rate-determining step (RDS) and reaction pathways. In acidic conditions where there is abundant hydronium serving as the proton source, the rate of the Volmer and Heyrovsky step exceeds that of the Tafel step.^5^ Consequently, the slowest Tafel step is the RDS, resulting in a theoretical Tafel slope of ∼30 mV/dec. In neutral and alkaline conditions, the Volmer step becomes the RDS because H_2_O replaces H_3_O^+^ as the proton source, which leads to a more sluggish water dissociation process.^6^ In this scenario, the theoretical Tafel slope is 120 mV/dec. In certain conditions, the Heyrovsky step could also behave as the RDS with a theoretical Tafel slope of 40 mV/dec. Thus, from the pure kinetic point of view, a Tafel slope between 30 and 120 mV/dec would be expected. However, Tafel slopes outside this range have been frequently reported with limited or no interpretation, raising considerable questions or doubts regarding the validity of the relevant analyses or claims.

A precise evaluation of the Tafel slope for a given catalyst in a given reaction condition is critical for determining the HER/HOR kinetics, deriving the RDS, and evaluating the underlying molecular mechanism. There are two widely used strategies for extracting the Tafel slopes from the HER polarization curves: (i) linear fitting of the overpotential vs logarithm of the HER kinetic current measured in the N_2_-purged electrolytes; and (ii) Bulter–Volmer (B–V) fitting of the HER and HOR kinetic currents in the H_2_-purged electrolytes.

Although it has been suggested that the linear fitting of polarization curve may not be an appropriate method due to the lack of a well-defined equilibrium potential in the N_2_-purged electrolytes,^1,7^ most of studies to date still employ the linear fitting method to extract the Tafel slopes for HER catalysts measured in the N_2_-purged electrolytes.^3,8−24^ Substantially different Tafel slopes have been reported even for the standard Pt/C catalysts. For example, in acidic conditions, Tafel slopes smaller than the theoretical limit of 30 mV/dec have been frequently reported without explanation.^14,23,25−28^ It is interesting to note that methods using a hydrogen pump with accelerated mass transport have shown a Tafel slope of approximately 120 mV/dec of Pt in the acidic condition, suggesting a Volmer step as the RDS in the acidic condition, which challenges the traditional interpretation of the Tafel step as RDS with 30 mV/dec Tafel slope.^7,29^ This further highlights the inconsistencies in Tafel slope values measured by different methodologies. Similarly, in the alkaline conditions, Tafel slopes ranging from 30 mV/dec to 60 mV/dec have been reported,^8−18,24^ which is far smaller than the theoretical Tafel slopes of 120 mV/dec expected for Volmer-step limited HER in alkaline electrolytes. Such inconsistencies raise questions about the validity of the Tafel analysis for characterizing the reaction kinetics. Although there have been suggestions that the deviations from the ideal model might originate from the diffusion-controlled HER behavior rather than kinetics control,^3^ a systematic and dedicated analysis is much needed to resolve the persistent inconsistencies in the literature.

To properly use the Tafel slope as a key kinetic parameter for characterizing the reaction kinetics and identifying the reaction mechanisms, it is important to closely evaluate the appropriateness and accuracy of the Tafel analysis methodology. Herein, we comprehensively analyze the uncertainties with the linear fitting method and discuss some potential issues with the B–V method. Our studies reveal that the Tafel slopes determined using the linear-fitting method are highly loading-dependent and potential-dependent and thus cannot be used as an unambiguous parameter for describing the HER kinetics of a given catalyst material. A systematic analysis further indicates that deviation from the ideal value can be largely ascribed to the locally trapped HER-generated H_2_ in the catalyst layer, which produces a non-negligible HOR current that more significantly offsets the HER current at lower HER overpotential and leads to an artificially lower Tafel slope, and suppresses the continued HER current growth at high overpotential and thus leads to an artificially higher Tafel slope. Such trapped H_2_ cannot be fully removed by using a rotating disc electrode with a vigorous rotation. The B–V method accounts for both HER and HOR current and could offer a better method for extracting the Tafel slope in the low overpotential regime, although the mass loading should be kept low enough to ensure a sufficient wide potential window of kinetic current for reliable fitting. We further note that the B–V equation cannot be readily used for describing the more complex HER catalysts with transition-metal decorated Pt surface (Pt/TM) due to the possible change of surface oxidation states and reaction mechanism within the fitting potential window.

## The Loading-Dependent Tafel Slopes

**Figure 1:**
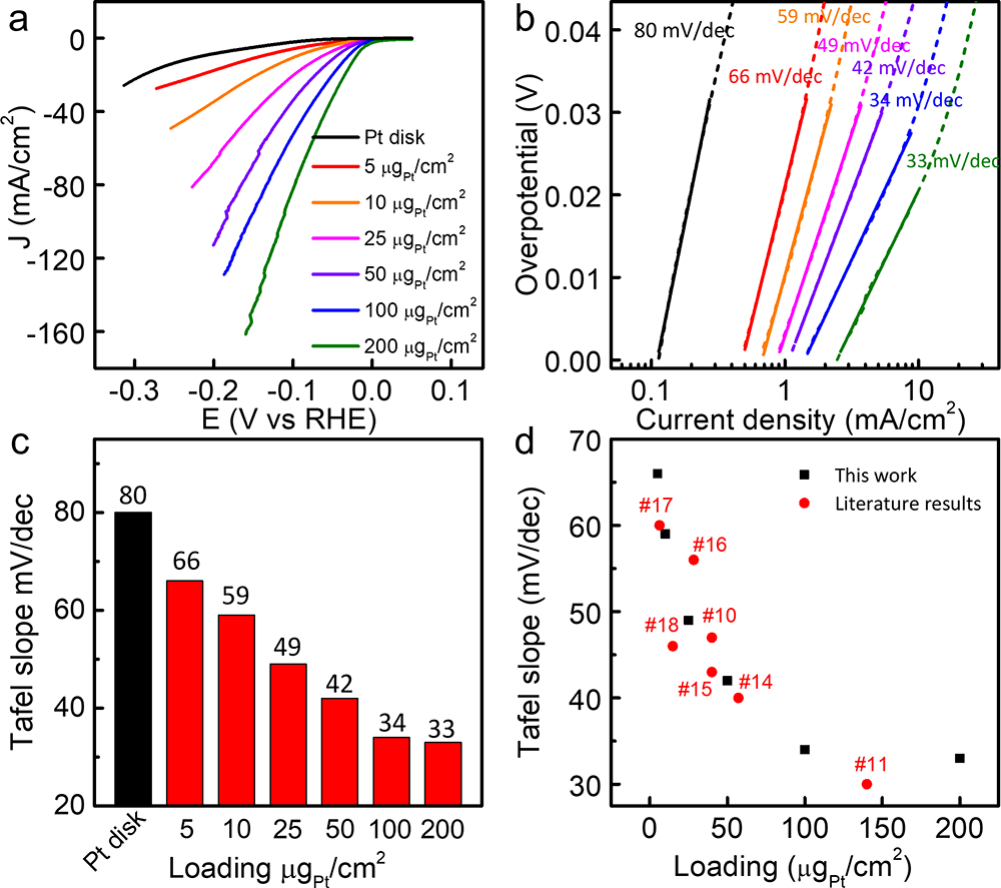
The loading-dependent Tafel slopes for HER on Pt/C. (a) HER polarization curves of Pt/C with 5, 10, 25, 50, 100, 200 μg_Pt_/cm^2^ and polycrystalline Pt disk electrode. (b) Tafel slope plot of Pt/C with 5, 10, 25, 50, 100, 200 μg_Pt_/cm^2^ and polycrystalline Pt disk electrode. (c) Summary of the Tafel slopes for HER on Pt/C with 5, 10, 25, 50, 100, 200 μg_Pt_/cm^2^ and polycrystalline disk Pt electrode and (d) comparison with literature values.

Figure 1a shows linear sweep voltammetry (LSV) curves in N_2_-purged 1 M KOH for the standard commercial Pt/C catalyst with different loadings ranging from 5 μg_Pt_/cm^2^ to 200 μg_Pt_/cm^2^, which covers most Pt mass loadings used in the literature. Using the linear fitting method (Figure 1b), the derived Tafel slopes exhibit a steady decrease from 66, 59, 49, 42, 34, to 33 mV/dec with the increasing Pt/C mass loading from 5, 10, 25, 50, 100, to 200 μg_Pt_/cm^2^, respectively (Figure 1c). A comparison of the Tafel slopes obtained in our study with those reported in the literature^10,11,14−18^ shows a generally consistent trend with the Pt mass loadings (Figure 1d), suggesting that the inconsistent Tafel slopes reported in the literature can be largely ascribed to the different Pt loadings. Notably, these Tafel slopes are all much smaller than the theoretically expected value of 120 mV/dec. A Tafel slope of 80 mV/dec was also observed on the Pt polycrystalline electrode (see black line or bar in Figure 1b,c), which excludes the possible contribution from the chemical property of the carbon black substrate. A similar loading-dependent Tafel slope can also be observed on commercial PtNi/C catalysts (Figure S1).

It has been suggested that the alkaline conditions are much more complex due to the coexistence of abundant hydrated cations and surface adsorbed hydroxyls (OH_ad_) at the Pt/electrolyte interface that may affect the local water structure and fundamentally modify the reaction pathways and kinetics.^30^ Studies have shown that the apparent HER activity of Pt increases from pH 7–14, suggesting that the reaction kinetics in the alkaline conditions may be different from that in the neutral conditions.^31^ It has also been reported that in alkaline conditions the step sites and surface OH_ad_ can accelerate the Volmer step, leading to a Tafel slope smaller than 120 mV/dec. However, these hypotheses cannot explain why the measured Tafel slopes are Pt-loading dependent.

## The Potential-Dependent Tafel Slopes

**Figure 2:**
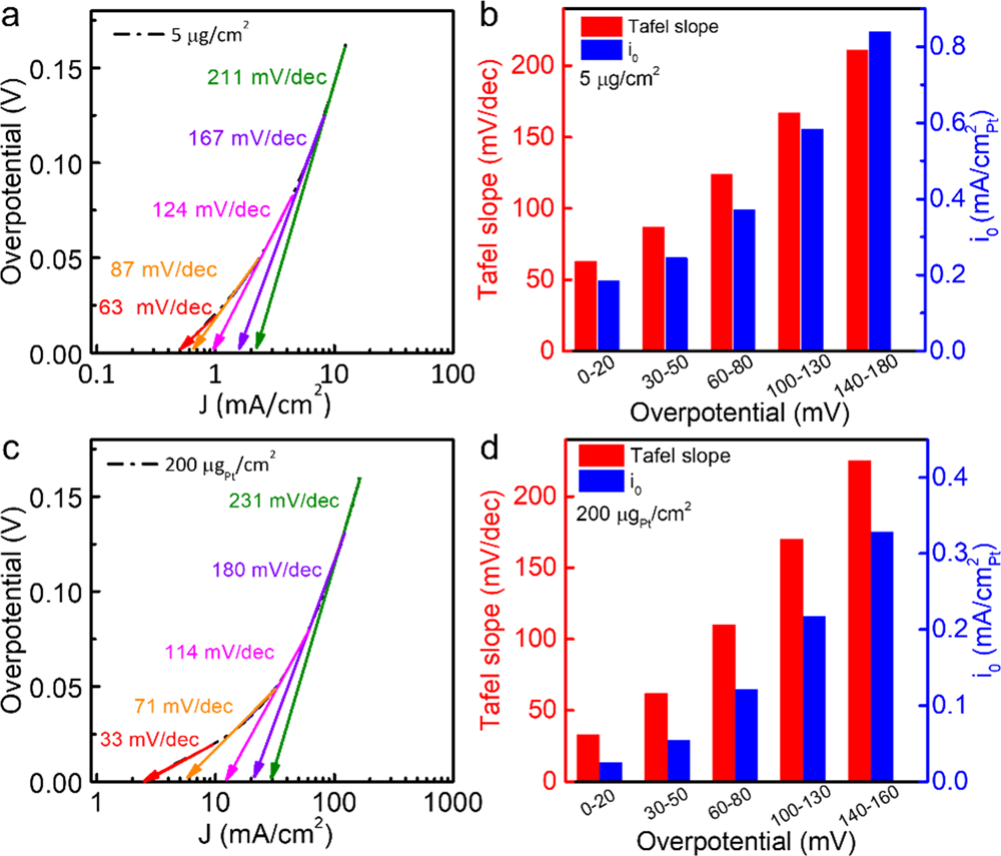
The potential-dependent Tafel slopes for HER on Pt/C. (a) Tafel plot of Pt at 5 μg_Pt_/cm^2^ loading. The Tafel slopes increase with an increased overpotential. (b) Tafel slopes obtained at different potential regions and the relevant exchange current density. Pt loading: 200 μg_Pt_/cm^2^. The *i*_0_ increases with an increased Tafel slope. (c) Tafel plot of Pt with 200 μg_Pt_/cm^2^ loading. The Tafel slopes increase with increased overpotential. (d) Tafel slopes obtained at different potential regions and the relevant exchange current densities. Pt loading: 200 μg_Pt_/cm^2^. The *i*_0_ increases with increased Tafel slope.

A more careful analysis further reveals that the Tafel slopes derived from the linear fitting method are also highly dependent on the exact overpotential range used for fitting. At a Pt loading of 5 μg_Pt_/cm^2^, the fitted Tafel slopes are 63, 87, 124, 167, and 211 mV/dec for the potential range of 0–20 mV, 30–50 mV, 60–80 mV, 100–130 mV, and 140–160 mV, respectively (Figure 2a,b). Similar phenomena are also observed at higher Pt loadings. At a loading of 200 μg_Pt_/cm^2^, the Tafel slopes fitted from 0 to 20 mV, 30–50 mV, 60–80 mV, 100–130 mV, and 140–160 mV are 33, 71, 114, 180, and 231 mV/dec, respectively (Figure 2c,d).

Moreover, such a potential-dependent Tafel slope brings up a critical problem when calculating the exchange current density (*i*_0_), which should be a fixed value representing the intrinsic activity at the thermodynamic equilibrium potential. The *i*_0_ can be extracted by extrapolating the linear regression line to the *X*-axis. Thus, a smaller Tafel slope mathematically leads to a smaller *i*_0_ (Figure 2a,c). Without appropriate criteria to select the kinetic region, it is difficult to obtain a meaningful Tafel slope or exchange current density *i*_0_ from the HER polarization curve. The kinetic information extracted using this approach is highly dependent on the exact experimental conditions and can be arbitrary and misleading for the understanding of reaction mechanisms when taken out of the context of the exact measurement conditions.

## The Role of H_2_ Diffusion in Tafel Analysis

**Figure 3:**
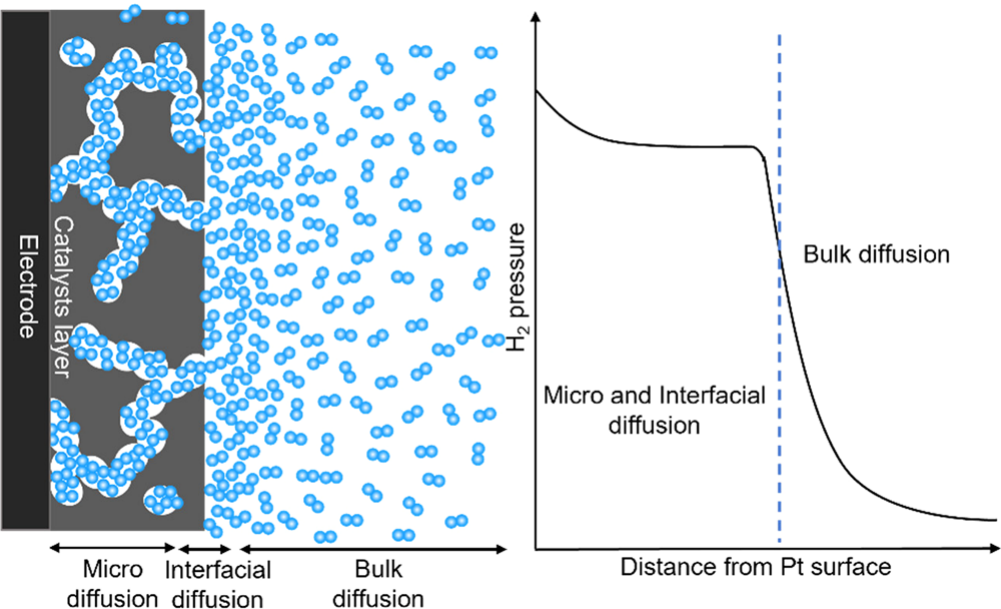
Illustration of microdiffusion, interfacial-diffusion, and bulk-diffusion. Due to sluggish microdiffusion and interfacial-diffusion, the HER-generated H_2_ accumulates in the catalyst layer, resulting in higher H_2_ local pressure that affects the apparent Tafel slope derivation.

During the HER process, the generated H_2_ on Pt nanoparticles’ surface needs to diffuse through the microchannels in the catalyst layer to the surface of the catalyst layer and eventually the bulk electrolyte. This diffusion process can be roughly separated into three components (Figure 3): (i) diffusion within the microchannels in the catalyst layer (microdiffusion), (ii) diffusion through the catalyst layer (interfacial-diffusion), and (iii) diffusion from the catalyst surface to the bulk electrolyte (bulk-diffusion).

If the H_2_ transport were solely dictated by the bulk-diffusion, the Tafel slopes derived from LSV curves with different Pt loading would surpass 120 mV/dec at the same geometric current density (current normalized by RDE geometric surface area). However, this is not the case. Our studies indicate that the Tafel slope of 200 μg_Pt_/cm^2^ (Figure 2c) surpasses 120 mV/dec at around 40–50 mA/cm^2^, while the Tafel slope of 5 μg_Pt_/cm^2^ (Figure 2a) has already surpassed 120 mV/dec at a much lower current density (3 mA/cm^2^). This observation suggests that, beyond bulk-diffusion, the microdiffusion and interfacial-diffusion play a more dominant role in hindering the growth of the HER current since the H_2_ microdiffusion and interfacial-diffusion are much slower than that of bulk-diffusion and may dictate the overall H_2_ mass transport process. More importantly, such microdiffusion and interfical-diffusion limitations are non-negligible even at a very low current density and a low overpotential, which makes it difficult to fully deconvolute the diffusion behavior from the kinetic behavior in extracting the Tafel slopes.

## Reoxidation of the HER-Generated H_2_

**Figure 4:**
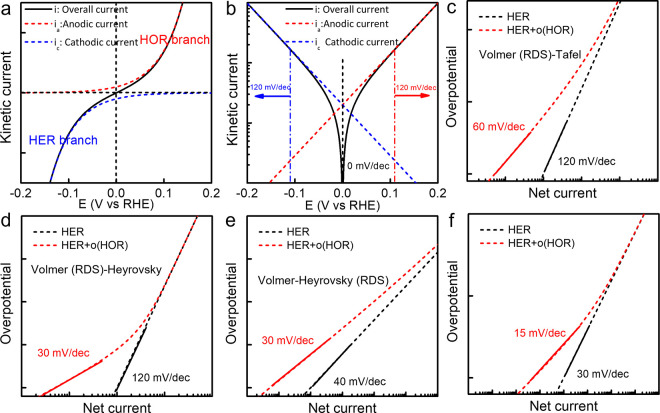
Effect of in situ reoxidation of H_2_ on the HER Tafel slope. (a) Relationship between HER current and HOR current and the net current. (b) The Tafel slope of HER and HOR branches near 0 V vs RHE are affected by the reverse reaction. Demonstration of the effect of H_2_ reoxidation on the measured Tafel of Pt/C (N_2_ saturated electrolyte) for (c) Volmer (RDS)-Tafel pathway, (d) Volmer (RDS)-Heyrovsky pathway, (e) Volmer-Heyrovsky (RDS) pathway, and (f) acidic condition.

To further understand the impact of H_2_ microdiffusion and interfacial-diffusion on the Tafel slopes extracted from the linear fitting method, we suggest a model that couples HER with partial reoxidation of the locally accumulated H_2_ to explain smaller than theoretical Tafel slopes observed near 0 V vs RHE. Considering the HER/HOR as a pair of reversible reactions, both the cathodic HER kinetic current (*i*_HER_) and the anodic HOR kinetics current (*i*_HOR_) scale exponentially with changing potential η (eqs 4 and 5, Figure 4a). Thus, we should expect a linear relationship between the η and Log(|*i*_HOR_| or |*i*_HER_|) and a constant HER Tafel slope throughout the entire potential range (Figure 4b, red and blue dash lines).

However, the net current (*i*_net_) is the sum of *i*_HER_ and *i*_HOR_ terms (eq 6 and Figure 4a), and thus the apparent Tafel slope is highly potential-dependent near 0 V vs RHE where HER and HOR have nearly comparable reaction rates. A straightforward model shows that the Tafel slopes would not reach a constant value until beyond ±120 mV vs RHE due to non-negligible contribution from the reverse reaction (Figure 4b). Therefore, in the H_2_ saturated electrolyte, linear fitting can only be used to accurately extract the Tafel slope above the 120 mV overpotential. In this regard, it seems that using 120 mV as the starting point to do linear fitting is a good criterion, even under N_2_ saturated electrolytes, in order to avoid impact from reoxidation of the HER-produced H_2_ in the catalyst layer. However, as discussed in the previous section, for Pt catalysts with high HER activity, the current density at around −120 mV vs RHE is already substantially affected by micro- and interfacial-diffusion issues, thus giving a Tafel slope exceeding the theoretical maximum value of 120 mV/dec regardless of the exact RDS (Figure S2). This gives no feasible potential window for reliable extraction of Tafel sloped using the linear fitting method.

In the literature, the linear fitting method has been commonly used for HER conducted under the N_2_ saturated electrolyte. This approach is only valid when there is no HOR taking place under N_2_ saturated electrolyte (blue curves Figure 4b). However, even under N_2_ saturated electrolyte, the HER-generated H_2_ could be trapped and accumulated in the catalyst layer due to the micro- and interfacial-diffusion limitations, creating an H_2_-rich microenvironment near the Pt nanoparticles’ surface, which can result in a non-negligible HOR current near 0 V vs RHE. Such minor HOR current can partly offset the HER current, which is more prominent at lower HER overpotential, leading to an apparently more rapid growth of the measured HER current (HER current + HOR current) going from 0 V vs RHE to more negative potential and thus an artificially lower Tafel slope.

## Microdiffusion Model for Interpreting Smaller than Theoretical Tafel Slopes

To quantiatively describe the role of the microdiffusion, interfacial-diffusion limitations, and the backward HOR in determining the measured Tafel slopes, we constructed an analytic model combining the HER, H_2_ diffusion, and HOR. Under the steady state, the amount of H_2_ produced from HER equals the amount of consumed H_2_ from the HOR and the amount of H_2_ that diffuses into the bulk electrolyte. Assuming the reaction follows the Volmer (RDS)-Tafel reaction pathway with the electron transfer coefficients α = β = 0.5, the *i*_HER_ and *i*_HOR_ could be written as
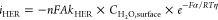
4
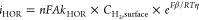
5

The net current *i*_net_ is the sum of *i*_HER_ and *i*_HOR_.
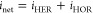
6
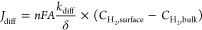
7

The H_2_ diffusion current *J*_diff_ describes the current behavior under the control of H_2_ diffusion from the active sites to the bulk electrolyte through the catalyst layer, including micro-, interfacial-, and bulk-diffusion. *C*_H_2_,surface_ and *C*_H_2_,bulk_ represent the H_2_ concentrations at the Pt surface and in the bulk electrolyte. *K*_diff_ is the diffusion coefficient and the δ is the thickness of the diffusion layer. *A* is the geometric surface area. *F* is the Faradaic constant, and *R* is the ideal gas constant. *T* is the temperature, and η is the potential.

With constant N_2_ purging, the *C*_H_2_,bulk_ = 0, we obtain
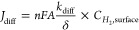
8Under the steady state, assuming that the , we have
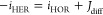
9and
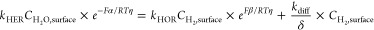
10
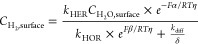
11Combining together, it gives
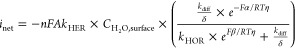
12
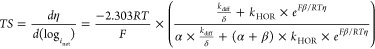
13Thus, when the ≫ *k*_*HOR*_, at 0 V vs RHE, we have
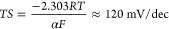
14When the *k*_HOR_≫ , at 0 V vs RHE, we have
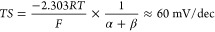
15

These analyses indicate that if the reaction follows the Volmer (RDS)-Tafel pathway, when the diffusion rate is much faster than the HOR rate, the measured Tafel slope approaches the theoretically expected value of 120 mV/dec. When the diffusion rate is much slower than the HOR rate, the measured Tafel slope could be as low as 60 mV/dec. Thus, possible Tafel slopes in the range of 60 and 120 mV/dec can be obtained from the simple linear fitting near 0 V vs RHE (Figure 4c). A higher Pt loading leads to a thicker catalyst layer with a smaller surface-to-volume ratio and a longer diffusion path for H_2_ to escape the catalyst layer, which results in a more sluggish H_2_ mass transport, an increased concentration of the locally trapped H_2_ in the catalyst layer, and thus a more prominent HOR current and apparently lower Tafel slope near the equilibrium potential. Similarly, if the reaction follows the Volmer (RDS)-Heyrovsky or Volmer-Heyrovsky (RDS) pathway, the lowest Tafel slope is approximately 30 mV (Figure 4d,e).

In acidic conditions, the HER kinetics are generally much faster. Thus, the mass transport limitation becomes an even more serious issue at a much lower Pt loading or much lower overpotential. For example, even with an ultralow Pt loading of 13 ng/cm^2^, the mass transport limitation still dominates the HER activity,^32^ which further complicates the identification of the well-defined RDS in acidic conditions.^3^ Nonetheless, although there is no well-defined expression for HER and HOR behaviors under acidic conditions, the non-negligible HOR current from reoxidation of the trapped H_2_ near the equilibrium potential could still lead to lower than the 30 mV/dec Tafel slopes frequently reported in acidic electrolytes. For example, in a simplified scenario with the assumption that both the HER and HOR are symmetric and limited by the Tafel step with a theoretical kinetic Tafel slope of 30 mV/dec, the apparent Tafel slope obtained from the linear fitting method can be lowered to 15 mV/dec if the reoxidation of locally generated H_2_ is considered (Figure 4f and Figure S3). It should be noted that the above model is based on the steady-state assumption. In an unsteady state where the amount of produced H_2_ is significantly larger than the amount of the H_2_ consumed by HOR and diffusion, an excessive supersaturation of H_2_ near the Pt surface could be expected,^33^ which would further amplify the HOR current at low overpotential and decrease the apparent HER Tafel slope to a value lower than the prediction based on steady-state model discussed above. Thus, the linear fitting method near 0 V vs RHE in the N_2_-purged condition could often lead to an artificially smaller Tafel slope and generate misleading conclusions for kinetic analysis.

Based on our hypothesis, for highly irreversible redox pairs such as ORR/OER, the kinetic slope of the OER Tafel slope in the kinetic region would be unaffected by the backward ORR and, consequently, loading-independent. To explore this point, we have conducted OER tests with Ir nanowires catalysts at various loadings, which showed consistent Tafel slopes of approximately ∼48 mV/dec with the loading varying from 10 μg/cm^2^ to 50 μg/cm^2^ (Figure S4). This observation further substantiates the robustness of our analyses and conclusions.

## B–V Fitting

To properly account for both the HER and HOR currents near the equilibrium potential, the fitting of HER/HOR kinetic current (*i*_*k*_) using the B–V equation (eq 16) may offer a more reliable approach to extract the Tafel slope.
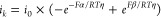
16
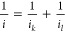
17
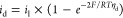
18
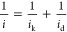
19

**Figure 5:**
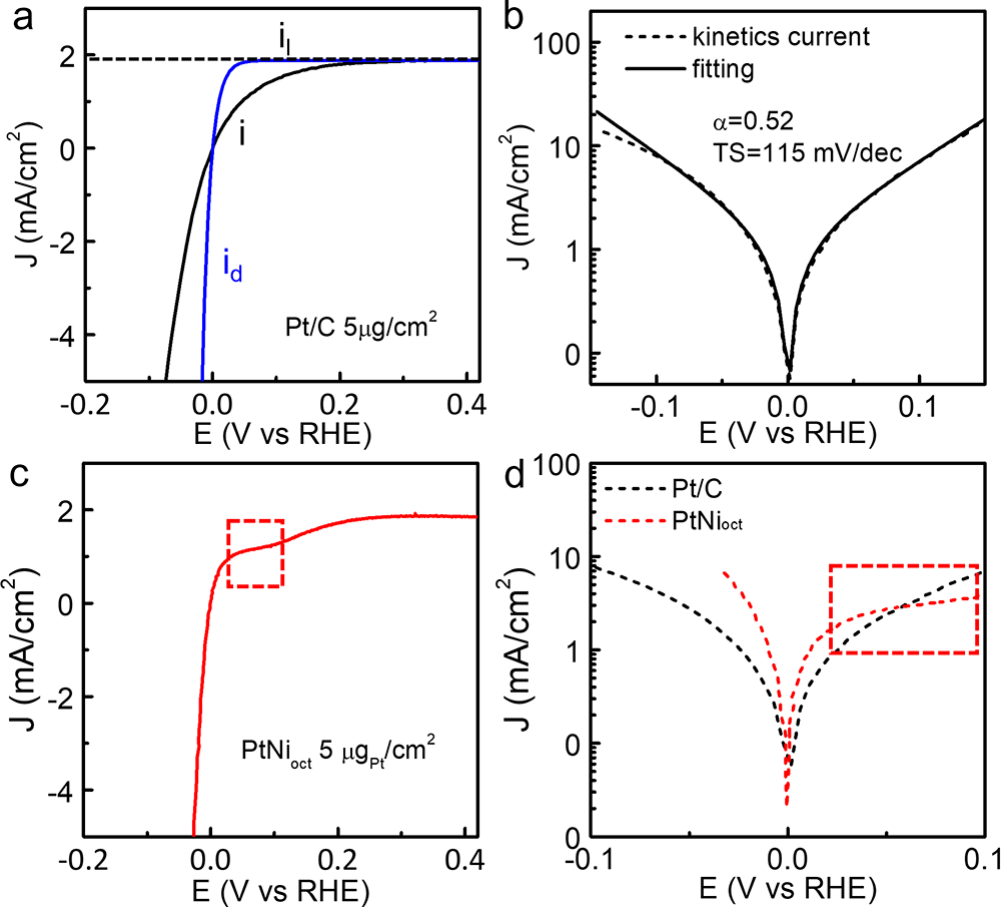
Bulter–Volmer equation to describe the HER/HOR kinetics. (a) The measured HER/HOR polarization curve (*i*), the diffusion current (*i*_d_), and the limiting current (*i*_l_) of Pt/C under H_2_ saturated 1 M KOH. (b) B–V fitting of HER/HOR kinetic current. (c) HER/HOR polarization curve of PtNi_oct_ under H_2_ saturated 1 M KOH shows a plateau region (highlighted by red dashed rectangle) after 0.02 V vs RHE. (d) Comparison of the HOR/HER kinetic currents for Pt/C and PtNi_oct_. The enhancement HOR by Ni-decorations gradually vanishes when the Ni is oxidized (highlighted by red dashed rectangle).

The Tafel slope and *i*_0_ can be further extracted from the fitted parameters. The *i*_k_ can be calculated from the overall current *i* and limiting current *i*_*l*_ using the irreversible Koutecký–Levich (K-L) equation (eq 17). With a rotating disc electrode, the HOR current increases exponentially with increasing overpotential but rapidly reaches a constant *i*_l_ due to the H_2_ mass transport limitations (from the bulk electrolyte to the Pt surface) (Figure 5a). Meanwhile, it was believed that the HER in the alkaline condition is not limited by the mass transport (however not always true, as pointed out in our analysis above), and hence, the overall current is the kinetic current. The combination of the HER/HOR kinetic current can then be used for B–V fitting. However, it was suggested that the diffusion current (*i*_d_, eq18), which is originated from the concentration overpotential (*η*_d_, caused by the concentration gradient of the reactants or the products in the bulk electrolyte and on the electrode surface due to the mass transport limitations) should be used instead of *i*_l_ to correct both the HER and HOR current using reversible K-L equation (eq 19).^3,34,35^ Based on these considerations, for pure Pt-based catalysts, extracting the alkaline HER/HOR kinetics using B–V fitting and the reversible K-L equation near the equilibrium potential under low Pt loading appears to be a reasonable strategy. For example, a fitting of the HER/HOR polarization curve in the 1 M KOH with the loading of 5 μg_Pt_/cm^2^ gives a Tafel slope of around 115 mV/dec (Figure 5b), which is close to the theoretically expected value of 120 mV/dec.

Furthermore, caution is advised when employing the B–V fitting method to describe the HER/HOR kinetics on Pt/TM surfaces. In contrast to the pure Pt surface where the HER and HOR are highly reversible and a well-defined B–V equation can be used to describe the mechanism, the HER and HOR kinetics on Pt/TM surfaces are far more complicated. For example, in contrast to the nearly symmetric HER/HOR characteristics observed in Pt/C, PtNi octahedra (PtNi_oct_) with a NiO decorated Pt (111) surface displays a rapidly plateauing HOE current at around 0.02 V vs RHE (Figure 5c). Although PtNi_oct_ displays notably higher HOR current than the Pt/C near 0 V vs RHE, this advantage diminishes above 0.05 V (Figure 5d). In this case, the conventional B–V equation cannot effectively describe the plateau region beyond 0.02 V in the HOR branch.

An essential requirement in applying B–V fitting is that the reaction mechanism and chemical states of the electrode surface should be potentially independent. However, this is not always the case. First, both the Pt surface charge and interfacial water structure are potential dependent.^5,36−38^ Moreover, while the Pt oxidation state is largely stable within the HER/HOR potential window, *in situ* XANES studies have shown that the Ni oxidation state may vary between Ni^0^ and Ni^2+^ within this potential regime.^39^ It was believed that the mixed Ni^0^/Ni^2+^ oxidation states play a key role in promoting the exchange of the surface OH_ad_ with the bulk electrolyte, thereby benefiting the HER/HOR kinetics, while the fully oxidized Ni^2+^ species would lose the ability to exchange the OH_ad_ with the electrolytes and therefore lose its capability of enhancing HOR.^39^ A similar plateau region was also observed on the Ru-decorated Pt surface where the decorated Ru^0^ loses its ability to enhance HOR after being oxidized to Ru^3+^,^40^ further indicating that the HER/HOR kinetics on Pt/TM surface is not as reversible as we used to believe. Therefore, the traditional mechanism that assumes a potential-independent chemical state may not be applicable for describing the HER/HOR on Pt/TM catalysts with potential dependent surface chemical states. This makes it difficult to use B–V fitting to resolve the HER/HOR kinetics of Pt catalysts and calls for the development of alternative analysis and interpretation methodologies.

## Conclusion

In summary, combining systematic experimental studies with analytical models, we reveal that the Tafel slopes extracted using the linear fitting method are highly loading- and potential-dependent, which is ascribed to the sluggish H_2_ diffusion in the catalyst layer. Our analysis indicates that the sluggish microdiffusion and interfacial-diffusion rather than the bulk-diffusion dictate the total H_2_ diffusion rate, resulting in a higher local-H_2_ pressure in the catalyst layer throughout the whole potential region. At low overpotential regime (−60–0 mV vs RHE), the non-negligible HOR current from the reoxidation of the trapped H_2_ partly offsets the HER current, leading to an underestimation of the HER Tafel slope. In the high overpotential regime (e.g., −60 to 120 mV vs RHE) where HOR becomes negligible, the mass transport limitations retards the continued growth of HER current with increasing overpotential, the linear fitting tends to give an overestimation of the Tafel slope. To address these complications and improve the reliability in Tafel slope analyses and comparisons, strategies that can help mitigate mass transport limitations are desirable, including

(1) Avoid excessive catalyst layer thickness to minimize the H_2_ mass transport resistance through the catalyst layer. We suggest a uniformly covered catalyst layer with a thickness smaller than 2 μm, with a Pt loading in the range of 10–50 ng/cm^2^ to reduce the H_2_ production rate and alleviate the H_2_ mass transport limitations.^41^
(2) Use carbon substrates conducive to H_2_ diffusion as the catalyst support. A superaerophobic (contact angle of the bubble with the surface is more than 150°) carbon substrate with a tunable pore size (e.g., surface-modified carbon nanotubes) that can enhance the local H_2_ diffusion and bubble removal could be an attractive candidate.^42^
(3) Adopt mass-transport-free methodology such as the hydrogen pump method to mitigate the mass transport limitation and extend the kinetic region for Tafel analysis.^29^
(4) Limit the range of overpotential (<50 mV) and current density (<2 mA/cm^2^) for Tafel analysis. The Tafel slopes measured above 50 mV or 2 mA/cm^2^ from the linear fitting method already exceed the theoretical maximum value of 120 mV/dec, indicating significant local mass transport limitations.

Despite the complications associated with H_2_ diffusion limitations, the linear fitting method, with strict control of the testing parameters such as catalyst loading and vigorous stirring, can still be applicable for internal comparison (same catalyst but different electrolyte pH, cations/anions, ionic strength, etc.) or qualitative evaluation of the relative activities. However, extreme caution should be exercised when making external comparisons among different catalysts or different studies to avoid misleading conclusions. Our analysis indicates that B–V fitting of the kinetic current under H_2_ saturated conditions is suitable for extracting the Tafel slope of pure Pt catalysts at low Pt loading conditions but is not necessarily unsuitable for determining the Tafel slopes for HER on the Pt/TM catalysts because of the potential-dependent chemical states and the irreversible HER/HOR kinetics.

## Data Availability

The data that support the plots within this paper and other findings of this study are available from the corresponding author upon reasonable request.

## Supporting Information Available

Experimental methods; H_2_ reoxidation models for Volmer (RDS)–Heyrovsky, Volmer–Heyrovsky (RDS) mechanisms, and acidic conditiosn; supporting figures for Tafel analysis of PtNi/C, Pt/C under N_2_/H_2_ conditions, Pt/C in the acidic conditions, and Ir nanowires (OER) (PDF)
Transparent Peer Review report available (PDF)
