# Supplementary material for: Unraveling and Resolving the Inconsistencies in Tafel Analysis for Hydrogen Evolution Reactions
Source: ACS Cent Sci. 2024 Feb 20;10(3):658–65. doi: 10.1021/acscentsci.3c01439 (PMC10979421; doi:10.1021/acscentsci.3c01439)
Supplement: Supplementary file 1 — oc3c01439_si_001.pdf [file oc3c01439_si_001.pdf]

Unraveling and Resolving the Inconsistencies in Tafel Analysis for Hydrogen Evolution Reactions

Chengzhang Wan[‡,†], Yansong Ling[‡], Sibo Wang[†], Heting Pu[‡,†], Yu Huang[‡,§,*], Xiangfeng Duan[†,*]

[†]*Department of Chemistry and Biochemistry, University of California, Los Angeles, California 90095, United States*

[‡]*Department of Materials Science and Engineering, University of California, Los Angeles, California 90095, United States*

[§]*California NanoSystems Institute, Los Angeles, California 90095, United States.*

[*]*Correspondence.* xduan@chem.ucla.edu, yhuang@seas.ucla.edu

Methods

No unexpected or unusually high safety hazards were encountered.

Chemicals: Commercial Pt/C catalyst (10 wt% Pt, and particle size ~ 2 nm) and commercial PtNi/C (20%) were purchased from Alfa Aesar. Potassium hydroxide (KOH) was purchased from Fisher Chemical. The deionized water (18 MΩ/cm) was obtained from an ultra-pure purification system (Milli-Q advantage A10). Nafion® 117 solution (~ 5%) was purchased from Sigma Aldrich.

Electrochemical Measurements: To obtain a homogeneous catalyst ink, 1 mg of Pt/C (10 wt%) was mixed with 1 mL ethanol and sonicated for 5 minutes. Then, 10 µL of Nafion 117 solution (5 wt%) was added to the solution. After sonication, a desired amount of the homogeneous ink was dropped onto a 5 mm diameter glassy carbon electrode (0.196 cm^2, Pine Research Instrumentation). The ink was dried under ambient air before electrochemical testing. The preparation of PtNi/C and $PtNi_{oct}/C$ electrodes follows the same protocol as preparing the Pt/C electrode.

All electrochemical tests were carried out in a three-electrode cell from Pine Research Instrumentation. The working electrode was a glassy carbon rotating disk electrode (RDE) coated with corresponding catalysts. The reference electrode was an Hg/HgO electrode from CH Instrument and was calibrated in 1.0 M KOH with saturated H_2. The HER polarization curve was scanned in 1.0 M KOH with N_2 purging or H_2 purging under 1600 r.p.m.

Derivation of H_2 reoxidation re-oxidation model for Volmer (RDS)-Heyrovsky mechanism

$$i_{HER} = -nFAk_{HER} \times C_{H_2O,\ surface} \times e^{-\frac{F\alpha}{RT}\eta} \tag{S1}$$

$$i_{HoR} = nFAk_{HoR} \times C_{H_2,\ surface} \times e^{\frac{F(1+\beta)}{RT}\eta} \tag{S2}$$

$$i_{net} = i_{HER} + i_{HOR} \tag{S3}$$

$$J_{diff} = nFA\frac{k_{diff}}{\delta} \times (C_{H_2,surface} - C_{H_2,\ bulk}) \text{ Equation} \tag{S4}$$

With N_2 purge, the $C_{H_2,\ bulk} = 0$,

and the $J_{diff} = nFA\frac{k_{diff}}{\delta} \times C_{H_2,surface}$ Equation (S5)

Under the steady state, assume that the $\frac{dC_{H_2,surface}}{dt} = 0$,

then $-i_{HER} = i_{HOR} + J_{diff}$ Equation (S6)

And $k_{HER}C_{H_2O,\ surface} \times e^{-\frac{F\alpha}{RT}\eta} = k_{HoR}C_{H_2,\ surface} \times e^{\frac{F(1+\beta)}{RT}\eta} + \frac{k_{diff}}{\delta} \times C_{H_2,surface}$ (S7)

$$C_{H_2,surface} = \frac{k_{HER}C_{H_2O,\ surface} \times e^{-\frac{F\alpha}{RT}\eta}}{k_{HOR} \times e^{\frac{F(1+\beta)}{RT}\eta} + \frac{k_{diff}}{\delta}} \tag{S8}$$

Combine together

$$i_{net} = -nFAk_{HER} \times C_{H_2O,\ surface} \times (\frac{\frac{k_{diff}}{\delta} \times e^{-\frac{F\alpha}{RT}\eta}}{k_{HOR} \times e^{\frac{F(1+\beta)}{RT}\eta} + \frac{k_{diff}}{\delta}}) \quad \text{(S9)}$$

$$TS = \frac{d\eta}{d(log_{i_{net}})} = \frac{-2.303RT}{F} \times (\frac{\frac{k_{diff}}{\delta} + k_{HOR} \times e^{\frac{F(1+\beta)}{RT}\eta}}{\alpha \times \frac{k_{diff}}{\delta} + (\alpha+1+\beta) \times k_{HOR} \times e^{\frac{F(1+\beta)}{RT}\eta}}) \quad \text{(S10)}$$

When the $\frac{k_{diff}}{\delta} \gg k_{HOR}$, at 0 V overpotential.

$$TS = \frac{-2.303RT}{\alpha F} \approx 120\ mV/dec \quad \text{(S11)}$$

When the $\frac{k_{diff}}{\delta} \ll k_{HOR}$, at 0 V overpotential.

$$TS \approx \frac{-2.303RT}{F} \times \frac{1}{\alpha+1+\beta} = 30\ mV/dec \quad \text{(S12)}$$

Derivation of H_2 re-oxidation model for Volmer-Heyrovsky (RDS) mechanism

$$i_{HER} = -nFAk_{HER} \times C_{H_2O,\ surface} \times e^{-\frac{F(\alpha+1)}{RT}\eta} \quad \text{(S13)}$$

$$i_{HoR} = nFAk_{HoR} \times C_{H_2,\ surface} \times e^{\frac{F\beta}{RT}\eta} \quad \text{(S14)}$$

$$i_{net} = i_{HER} + i_{HOR} \quad \text{(S15)}$$

$$J_{diff} = nFA\frac{k_{diff}}{\delta} \times (C_{H_2,surface} - C_{H_2,\ bulk}) \quad \text{(S16)}$$

With N_2 purge, the $C_{H_2,\ bulk} = 0$, and the $J_{diff} = nFA\frac{k_{diff}}{\delta} \times C_{H_2,surface}$ (S17)

Under the steady state, assume that the $\frac{dC_{H_2,surface}}{dt} = 0$, then $-i_{HER} = i_{HOR} + J_{diff}$ (S18)

And $k_{HER}C_{H_2O,\ surface} \times e^{-\frac{F(\alpha+1)}{RT}\eta} = k_{HoR}C_{H_2,\ surface} \times e^{\frac{F\beta}{RT}\eta} + \frac{k_{diff}}{\delta} \times C_{H_2,surface}$ (S19)

$$C_{H_2,surface} = \frac{k_{HER}C_{H_2O,\ surface} \times e^{-\frac{F(\alpha+1)}{RT}\eta}}{k_{HOR} \times e^{\frac{F\beta}{RT}\eta} + \frac{k_{diff}}{\delta}} \quad \text{(S20)}$$

Combine together

$$i_{net} = -nFAk_{HER} \times C_{H_2O,\ surface} \times (\frac{\frac{k_{diff}}{\delta} \times e^{-\frac{F(\alpha+1)}{RT}\eta}}{k_{HOR} \times e^{\frac{F\beta}{RT}\eta} + \frac{k_{diff}}{\delta}}) \quad \text{(S21)}$$

$$TS = \frac{d\eta}{d(log_{i_{net}})} = \frac{-2.303RT}{F} \times (\frac{\frac{k_{diff}}{\delta} + k_{HOR} \times e^{\frac{F\beta}{RT}\eta}}{(\alpha+1) \times \frac{k_{diff}}{\delta} + (\alpha+1+\beta) \times k_{HOR} \times e^{\frac{F\beta}{RT}\eta}}) \quad \text{(S22)}$$

When the $\frac{k_{diff}}{\delta} \gg k_{HOR}$, at 0 V overpotential.

$$TS = \frac{-2.303RT}{(1+\alpha)F} \approx 40\ mV/dec \quad (S23)$$

When the $\frac{k_{diff}}{\delta} \ll k_{HOR}$, at 0 V overpotential.

$$TS = \frac{-2.303RT}{F} \times \frac{1}{\alpha+1+\beta} \approx 30\ mV/dec \quad (S24)$$

Derivation of H_2 re-oxidation model in the acidic condition (see Figure S3 for detailed discussion on the assumption).

$$i_{HER} = -nFAk_{HER} \times C_{H_2O,\ surface} \times e^{-\frac{2F}{RT}\eta} \quad (S25)$$

$$i_{HoR} = nFAk_{HoR} \times C_{H_2,\ surface} \times e^{\frac{2F}{RT}\eta} \quad (S26)$$

$$i_{net} = i_{HER} + i_{HOR} \quad (S27)$$

$$J_{diff} = nFA\frac{k_{diff}}{\delta} \times (C_{H_2, surface} - C_{H_2,\ bulk}) \quad (S28)$$

With N_2 purge, the $C_{H_2,\ bulk} = 0$, and the $J_{diff} = nFA\frac{k_{diff}}{\delta} \times C_{H_2, surface}$ (S29)

Under the steady state, assume that the $\frac{dC_{H_2,surface}}{dt} = 0$, then $-i_{HER} = i_{HOR} + J_{diff}$ (S30)

And $k_{HER}C_{H_2O,\ surface} \times e^{-\frac{2F}{RT}\eta} = k_{HoR}C_{H_2,\ surface} \times e^{\frac{2F}{RT}\eta} + \frac{k_{diff}}{\delta} \times C_{H_2, surface}$ (S31)

$$C_{H_2, surface} = \frac{k_{HER}C_{H_2O,\ surface} \times e^{-\frac{2F}{RT}\eta}}{k_{HOR} \times e^{\frac{2F}{RT}\eta} + \frac{k_{diff}}{\delta}} \quad (S32)$$

Combine together

$$i_{net} = -nFAk_{HER} \times C_{H_2O,\ surface} \times \left(\frac{\frac{k_{diff}}{\delta} \times e^{-\frac{2F}{RT}\eta}}{k_{HOR} \times e^{\frac{2F}{RT}\eta} + \frac{k_{diff}}{\delta}}\right) \quad (S33)$$

$$TS = \frac{d\eta}{d(log_{i_{net}})} = \frac{-2.303RT}{F} \times \left(\frac{\frac{k_{diff}}{\delta} + k_{HOR} \times e^{\frac{2F}{RT}\eta}}{(2 \times \frac{k_{diff}}{\delta} + 4 \times k_{HOR} \times e^{\frac{2F}{RT}\eta}}\right) \quad (S34)$$

When the $\frac{k_{diff}}{\delta} \gg k_{HOR}$, at 0 V overpotential.

$$TS = \frac{-2.303RT}{2F} \approx 30\ mV/dec \quad (S35)$$

When the $\frac{k_{diff}}{\delta} \ll k_{HOR}$, at 0 V overpotential.

$$TS = \frac{-2.303RT}{F} \times \frac{1}{4} \approx 15\ mV/dec \quad (S36)$$

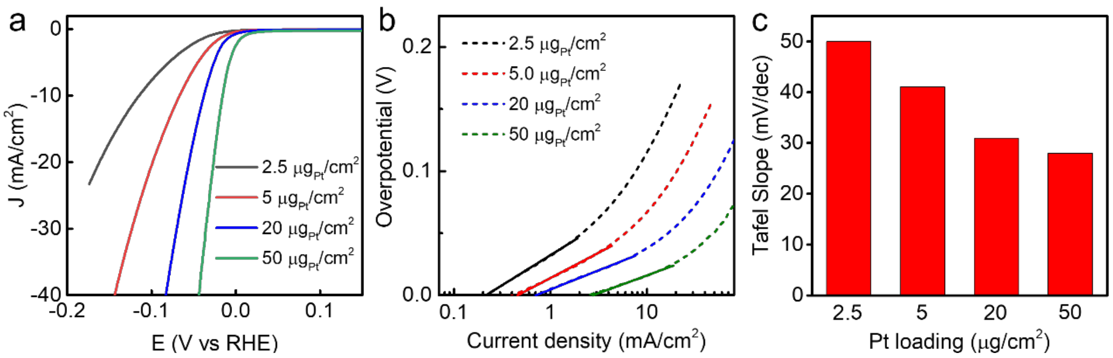

Figure S1. Loading-dependent Tafel slopes of PtNi/C. (a) HER Polarization curves of PtNi/C. (b) and (c) Tafel slopes measured by the linear fitting method. Similar to Pt/C, the PtNi/C also possesses loading-dependent Tafel slopes, indicating that it is a general phenomenon on both pure Pt and modified Pt surfaces. With Pt loading from 2.5 μg/cm^2 to 50 μg/cm^2, the Tafel slopes decrease from 50 mV/dec to even lower than 30 mV/dec. The lower than 30 mV/dec Tafel slope cannot be explained by any current reaction mechanism, and may be ascribed to the reoxidation of HER generated H_2.

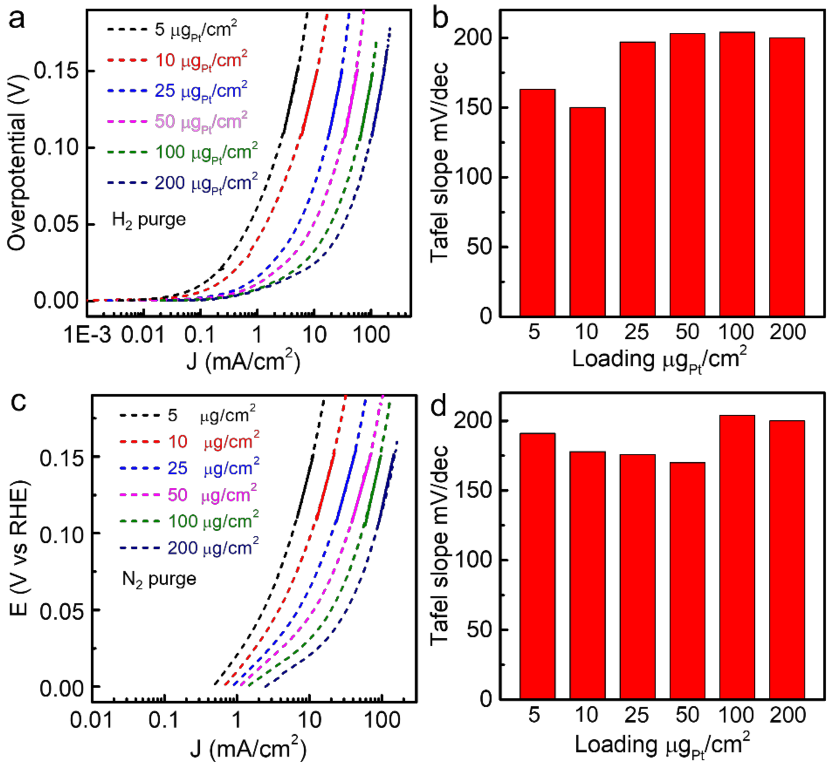

Figure S2. Tafel slopes of Pt/C exceed 120 mV/dec at -120 mV vs RHE under both H_2 and N_2 saturated electrolyte. (a) HER Polarization curve of Pt/C under H_2. (b) Tafel slope of Pt/C under H_2. (c) HER Polarization curve of Pt/C under N_2. (d) Tafel slope of Pt/C under N_2. Theoretically, the effect of the backward reaction on the linearly-fitted Tafel slope will disappear at the potential below -120 mV vs RHE. However, due to the H_2 local diffusion problem, the Tafel slopes at around -120 mV vs RHE have already significantly surpassed the maximum theoretical value of 120 mV/dec, in both N_2 and H_2 saturated condition for the whole loading range. This gives no feasible potential window for reliable extraction of Tafel slopes using the linear fitting approach.

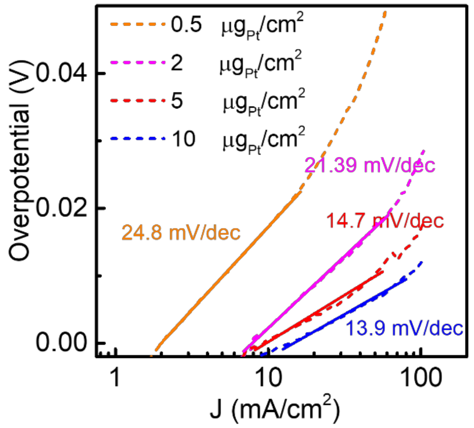

Figure S3. Tafel slope of Pt/C with different loading under N_2 purged acidic condition where the HER kinetics should be controlled by Volmer-Tafel (RDS) pathway or diffusion overpotential. All the measured Tafel slope is below the minimum theoretical value of 30 mV/dec, which could be ascribed to the reoxidation of locally trapped H_2, It is noted that the exact RDS in the acidic condition remains a topic of considerable debate. Methods using hydrogen pump with low mass transport resistances have shown a Tafel slope of approximately 120 mV/dec of Pt in the acidic condition,[1, 2] suggesting a Volmer step RDS in the acidic condition, which is at odd with traditional Tafel step RDS with 30 mV/dec Tafel slope. The 30 mV/dec Tafel slope can also be explained by the diffusion-controlled HER behavior in the acidic condition rather than a Tafel step RDS kinetics.[3] Additionally, due to the fact that no electron transfer is included in the Tafel step, the HOR with Tafel step as RDS is supposed to be potential independent. However, apparent potential-dependence can be observed in the acidic HOR branch.[4] Therefore, it is non-trivial to set up an exclusive equation for acidic condition. For a simplicity, the results in Figure 4f are derived by assuming the symmetric factor to be 2 for both HER and HOR branches for illustrating the effect of the H_2 reoxidation in Tafel derivation.

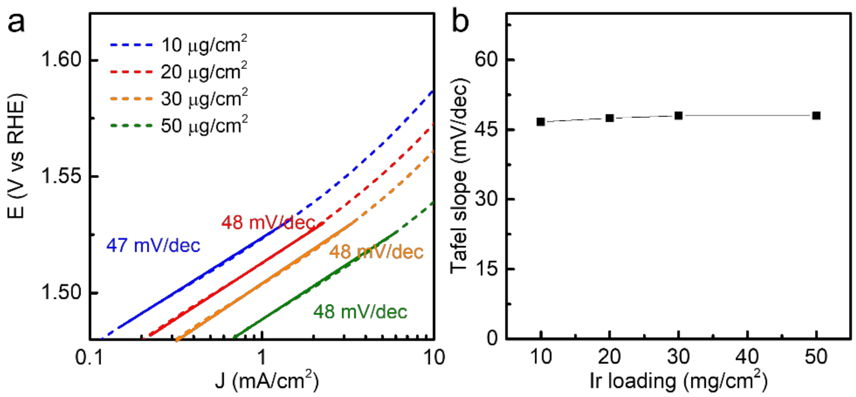

Figure S4. Evaluation of OER Tafel slopes for Ir nanowires catalysts at different loadings. (a) LSV and linear fitted Tafel slopes near onset potential. (b) Tafel slopes vs Ir loading. Different from HER which showed loading-dependent Tafel slopes due to the highly reversible backward HOR reaction, the Tafel slope of irreversible OER is loading-independent, further strengthening our theory that the deviation of HER Tafel slope from the theoretical value originated from the backward HOR of the locally trapped H_2.

References

1. Neyerlin, K. C.; Gu, W.; Jorne, J.; Gasteiger, H. A., Study of the Exchange Current Density for the Hydrogen Oxidation and Evolution Reactions. *Journal of The Electrochemical Society* **2007,** *154* (7), B631.
2. Durst, J.; Simon, C.; Hasché, F.; Gasteiger, H. A., Hydrogen Oxidation and Evolution Reaction Kinetics on Carbon Supported Pt, Ir, Rh, and Pd Electrocatalysts in Acidic Media. *Journal of The Electrochemical Society* **2015,** *162* (1), F190.
3. Zheng, J.; Yan, Y.; Xu, B., Correcting the Hydrogen Diffusion Limitation in Rotating Disk Electrode Measurements of Hydrogen Evolution Reaction Kinetics. *Journal of The Electrochemical Society* **2015,** *162* (14), F1470.
4. Prats, H.; Chan, K., The determination of the HOR/HER reaction mechanism from experimental kinetic data. *Physical Chemistry Chemical Physics* **2021,** *23* (48), 27150-27158.
