# Supplementary material for: Unraveling and Resolving the Inconsistencies in Tafel Analysis for Hydrogen Evolution Reactions
Source: ACS Cent Sci. 2024 Feb 20;10(3):658–65. doi: 10.1021/acscentsci.3c01439 (PMC10979421; doi:10.1021/acscentsci.3c01439)
Supplement: Supplementary file 2 — oc3c01439_si_002.pdf [file oc3c01439_si_002.pdf]

oc-2023-014392.R1

Name: Peer Review Information for "Unraveling and Resolving the Inconsistencies in Tafel Analysis for Hydrogen Evolution Reactions"

First Round of Reviewer Comments

Reviewer: 1

Comments to the Author

The manuscript by Wan et al. provides a clear explanation for the discrepancies in the numerical values between Tafel slope tests and ideal model calculations in RDE testing of HER reactions. The observed deviation in fitting Tafel slope at low current density is primarily attributed to H2 enrichment within the catalytic layer, leading to the occurrence of the reverse HOR reaction and thereby reducing the Tafel slope, resulting in erroneous outcomes. Further investigation into the dynamics of transition metal-modified Pt surfaces indicates disparities between the kinetics of Pt with and without transition metal modifications. The viewpoint of paper effectively addresses previous uncertainties regarding Tafel slopes and loading mentioned in the literature.

Overall, the paper is reasonably well-written and appealing to the ACS Central Science audience. However, the overall complexity of the work is relatively simplified. The latter part of the paper employs some basic knowledge of electrode kinetics and uses theory to support the earlier points. Unfortunately, it is still challenging for us to definitively confirm that the testing process is necessarily influenced as postulated by the authors. This represents a necessary but insufficient condition.

I recommend publication after addressing the following issues:

(1) The situation where different catalyst loading results in different Tafel slopes should not be limited to HER reactions only. Following the author's reasoning, if there is no reverse reaction, the Tafel slope calculation should not exhibit errors. Can the authors provide evidence from other reactions such as OER to demonstrate the impact of reverse reactions within the catalytic layer, ensuring the reliability of the author's conclusions?

(2) There are some errors in the manuscript, such as "Tafellopes" in the abstract and "μg" written as "mg" in Figure S1C.

(3) The essence of Tafel slope is to express the catalytic intrinsic activity. Concerning Figure 2 and the mention in the text about fitting Tafel slope at high current density, this practice is not advocated. Because, as we all know, at high current density, the generation of internal bubbles brings mass transfer potential, thereby altering the Tafel slope.

Reviewer: 2

Comments to the Author

This study reveals that the Tafel slopes derived from the linear fitting method are loading-dependent, potential-dependent, and frequently deviate substantially from ideal theoretical models. It attributes this issue to the impact of the hydrogen oxidation reaction (HOR) current generated by the localized trapping of H2 during the hydrogen evolution reactions (HER) by the catalyst layer. However, before this paper could be considered for publication, some issues should be well addressed.

1. The article states that the Butler-Volmer equation cannot easily describe complex transition metal-modified HER catalysts. However, transition metal-modified catalysts are predominant among low-cost HER catalysts. Could the author elaborate on how the B-V equation and Tafel slope apply to these transition metal HER catalysts? Could the author explain their ideas on this matter?

2. The article extensively discusses the drawbacks of current Tafel slope testing methods but doesn't mention how to improve them. Could you provide some additional information about possible improvements or general ideas to enhance these methods?

3. In the section "The loading-dependent Tafel slopes," could you supplement an explanation of why the loading amount affects Tafel slope?

Author's Response to Peer Review Comments:

Reviewer(s)' Comments to Author:

Reviewer: 1

Recommendation: Publish in ACS Central Science after minor revisions noted.

Comments:
The manuscript by Wan et al. provides a clear explanation for the discrepancies in the numerical values between Tafel slope tests and ideal model calculations in RDE testing of HER reactions. The observed deviation in fitting Tafel slope at low current density is primarily attributed to H2 enrichment within the catalytic layer, leading to the occurrence of the reverse HOR reaction and thereby reducing the Tafel slope, resulting in erroneous outcomes. Further investigation into the dynamics of transition metal-modified Pt surfaces indicates disparities between the kinetics of Pt with and without transition metal modifications. The viewpoint of paper effectively addresses previous uncertainties regarding Tafel slopes and loading mentioned in the literature. Overall, the paper is reasonably well-written and appealing to the ACS Central Science audience. However, the overall complexity of the work is relatively simplified. The latter part of the paper employs some basic knowledge of electrode kinetics and uses theory to support the earlier points. Unfortunately, it is still challenging for us to definitively confirm that the

testing process is necessarily influenced as postulated by the authors. This represents a necessary but insufficient condition.

I recommend publication after addressing the following issues:

Response: We are grateful for the reviewer's acknowledgment of our efforts in elucidating the origins of uncertainty and deviation in the current HER Tafel analysis, and appreciate the constructive comments raised. We welcome the opportunity to address these questions and describe our revisions accordingly.

(1) The situation where different catalyst loading results in different Tafel slopes should not be limited to HER reactions only. Following the author's reasoning, if there is no reverse reaction, the Tafel slope calculation should not exhibit errors. Can the authors provide evidence from other reactions such as OER to demonstrate the impact of reverse reactions within the catalytic layer, ensuring the reliability of the author's conclusions?

Response: We appreciate the reviewer's suggestion on the OER test. Based on our hypothesis, for highly irreversible redox pairs such as ORR/OER, the OER Tafel slope in the kinetic region should be unaffected by the backward ORR and, consequently, loading-independent. To explore this point, we have conducted OER tests with Ir nanowires catalysts at various loadings, which showed consistent Tafel slopes of ~48 mV/dec with the loading varies from 10 μg/cm^2 to 50 μg/cm^2 (Fig. R1). This observation further substantiates the robustness of our analyses and conclusions. Fig. R1 has been incorporated into the supporting information as Fig. S4, accompanied by a relevant discussion in the revised manuscript on page 13.

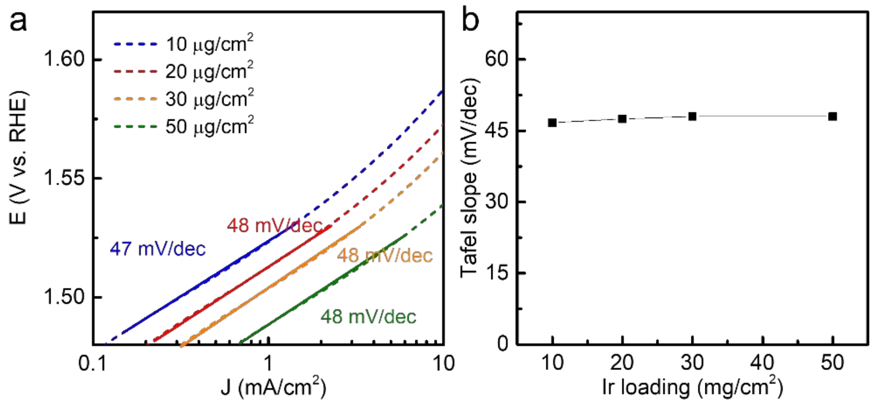

Figure R1. Evaluation of OER Tafel slopes for Ir nanowires catalysts at different loadings. (a) LSV and linear fitted Tafel slopes near onset potential. (b) Tafel slopes vs. Ir loading. Different from HER which showed loading-dependent Tafel slopes due to the highly reversible backward HOR reaction, the Tafel slope of irreversible OER is loading-independent, further strengthening our theory that the deviation of HER Tafel slope from the theoretical value originated from the backward HOR of the locally trapped H_2.

(2) There are some errors in the manuscript, such as "Tafellopes" in the abstract and "μg" written as "mg" in Figure S1C.

Response: Thanks, and we have revised all the typos in the main text and SI.

(3) The essence of Tafel slope is to express the catalytic intrinsic activity. Concerning Figure 2 and the mention in the text about fitting Tafel slope at high current density, this practice is not advocated. Because, as we all know, at high current density, the generation of internal bubbles brings mass transfer potential, thereby altering the Tafel slope.

Response: We fully concur with the reviewer regarding the impact of internal bubble generation on mass transport potential and the Tafel slope, as emphasized in our central argument illustrated in Fig. 2. We note there is a lack of well-defined potential regions for fitting the Tafel slope in the literature. An excessive overpotential and high current density aggravates the mass transport overpotential in the analysis, which could lead to artificially large Tafel slopes and artificially elevated exchange current density values. To underscore the inaccuracy of such practices, we explicitly demonstrate the impact of such practices in our paper, aiming to raise awareness of these common missteps within the research community.

Reviewer: 2

Recommendation: Reconsider after major revisions noted.

Comments:

This study reveals that the Tafel slopes derived from the linear fitting method are loading-dependent, potential-dependent, and frequently deviate substantially from ideal theoretical models. It attributes this issue to the impact of the hydrogen oxidation reaction (HOR) current generated by the localized trapping of H_2 during the hydrogen evolution reactions (HER) by the catalyst layer. However, before this paper could be considered for publication, some issues should be well addressed.

Response: We appreciate the reviewer's recognition of our work. A more detailed response to each question is provided below.

1. The article states that the Butler-Volmer equation cannot easily describe complex transition metal-modified HER catalysts. However, transition metalmodified catalysts are predominant among low-cost HER catalysts. Could the author elaborate on how the B-V equation and Tafel slope apply to these transition metal HER catalysts? Could the author explain their ideas on this matter?

Response: We appreciate the insightful question. We hypothesize that the oxidation state of the decorated transition metals might influence the concentration/distribution of surface-adsorbed oxygen in water, which could fundamentally alter the HER/HOR pathway at a slightly different potential. Limiting B-V fitting potential range within ±10 mV Vs. RHE seems reasonable for mitigating the complexity at higher potentials where transition metals start to be oxidized. We have an ongoing project aiming to unravel the underlying mechanism using *in-situ* characterizations, which is beyond the scope of the current study and will be reported in a separate dedicated study in the future.

2. The article extensively discusses the drawbacks of current Tafel slope testing methods but doesn't mention how to improve them. Could you provide some additional information about possible improvements or general ideas to enhance these methods?

Response: We thank the reviewer's comments. Enhancing Tafel slope testing methods involves addressing the identified drawbacks and implementing improvements. This work primarily focuses on elucidating the origin of the uncertainties in the frequently used Tafel slope analysis and raising awareness within the community. We note that it is not trivial to provide a universal solution to all the issues discussed in the current study. In general, strategies that can help mitigate mass transport limitations could be beneficial. Motivated by the reviewer's comments, we have further outlined some general considerations for improving reliability in Tafel slope analysis and comparisons (also in the conclusion part on page 16):

1) Avoid excessive catalyst layer thickness to minimize the H_2 mass transport resistance through the catalyst layer. We suggest a uniformly covered catalyst layer with a thickness smaller than 2 μm, with a Pt loading in the range of 10-50 ng/cm^2 to reduce the H_2 production rate and alleviate the H_2 mass transport limitations (Int. J. Hydrogen Energy. 2016, 41, 7568-7581).
2) Use carbon substrates conducive to H_2 diffusion as the catalyst support. Superaerophobic (contact angle of the bubble with the surface is more than 150°) carbon substrate with a tunable pore size (e.g., surface-modified carbon nanotubes) that can enhance the local H_2 diffusion and bubble removal could be an attractive candidate (J. Mater. Chem. A, 2022, 10, 5147-5173)
3) Adopt mass-transport-free methodology such as the hydrogen pump method (J. Electrochem. Soc. 2007, 154, B631) to mitigate the mass transport limitation and extend the kinetic region for Tafel analysis.
4) Limit the range of overpotential (< 50 mV) and current density (< 2 mA/cm^2) for Tafel analysis. The Tafel slopes measured above 50 mV or 2 mA/cm^2 from the linear fitting method already exceed the theoretical maximum value of 120 mV/dec, indicating significant local mass transport limitations.

3. In the section "The loading-dependent Tafel slopes," could you supplement an explanation of why the loading amount affects Tafel slope?

Response: We appreciate the reviewer's suggestions. For a given electrode size (diameter), the catalyst loading amount directly determines the thickness of the catalyst layer, which dictates the diffusion of H_2 out of the catalyst layer. In the section “Micro-diffusion model for interpreting smaller than theoretical Tafel slopes” (page 12 line 6), we revised to more clearly explain why the loading amount matters, “A higher Pt loading leads to a thicker catalyst layer with a smaller surface-to-volume ratio and a longer diffusion path for H_2 to escape the catalyst layer, which results in a more sluggish H_2 mass transport, an increased concentration of the locally trapped H_2 in the catalyst layer, and thus a more prominent HOR current and an apparently lower Tafel slopes near the equilibrium potential.”
